# Feasibility of low-cost particle sensor types in long-term indoor air pollution health studies after repeated calibration, 2019–2021

**DOI:** 10.1038/s41598-022-18200-0

**Published:** 2022-08-26

**Authors:** Elle Anastasiou, M. J. Ruzmyn Vilcassim, John Adragna, Emily Gill, Albert Tovar, Lorna E. Thorpe, Terry Gordon

**Affiliations:** 1 Department of Population Health, New York University Grossman School of Medicine, 180 Madison Avenue, New York, NY 10016 USA; 2 Department of Environmental Science, New York University Grossman School of Medicine, 341 East 25th Street, New York, NY 10010 USA; 3 Department of Environmental Health Sciences, University of Alabama at Birmingham School of Public Health, Birmingham, AL 205-934-8927 USA

**Keywords:** Biological techniques, Environmental sciences, Environmental social sciences, Energy science and technology, Materials science

## Abstract

Previous studies have explored using calibrated low-cost particulate matter (PM) sensors, but important research gaps remain regarding long-term performance and reliability. Evaluate longitudinal performance of low-cost particle sensors by measuring sensor performance changes over 2 years of use. 51 low-cost particle sensors (Airbeam 1 N = 29; Airbeam 2 N = 22) were calibrated four times over a 2-year timeframe between 2019 and 2021. Cigarette smoke-specific calibration curves for Airbeam 1 and 2 PM sensors were created by directly comparing simultaneous 1-min readings of a Thermo Scientific Personal DataRAM PDR-1500 unit with a 2.5 µm inlet. Inter-sensor variability in calibration coefficient was high, particularly in Airbeam 1 sensors at study initiation. Calibration coefficients for both sensor types trended downwards over time to < 1 at final calibration timepoint [Airbeam 1 Mean (SD) = 0.87 (0.20); Airbeam 2 Mean (SD) = 0.96 (0.27)]. We lost more Airbeam 1 sensors (N = 27 out of 56, failure rate 48.2%) than Airbeam 2 (N = 2 out of 24, failure rate 8.3%) due to electronics, battery, or data output issues. Evidence suggests degradation over time might depend more on particle sensor type, rather than individual usage. Repeated calibrations of low-cost particle sensors may increase confidence in reported PM levels in longitudinal indoor air pollution studies.

## Introduction

Studies of air pollution-associated health impacts often require measuring ambient concentrations of air pollutants. While monitoring of PM_2.5_ concentrations has contributed to understanding and reducing ambient PM_2.5_ to improve air quality standards, rigorous measurement of indoor air pollution remains a challenge.

Conventionally, the measurement of ambient PM_2.5_ concentrations requires either a labor-intensive gravimetric filter-based method with size-specific inlets, or sophisticated and manufacturer-calibrated real-time instruments. Such equipment is expensive and not readily portable, thus limiting the number of locations that can be sampled within a given time. Central monitoring networks that use advanced instruments utilizing gravimetry, light scattering, or beta attenuation have been mounted by states and federal agencies to address efforts to achieve federal national ambient air quality standards (NAAQS). Use of central monitoring is essential for monitoring PM_2.5_ exposures within the microenvironments of cities, in contrast, personal monitoring is considered the optimal approach to assess an individual’s exposure levels to PM_2.5_^1,2^.

Until recently, monitoring indoor settings at a high spatial and temporal resolution was impractical due to the cost, size, and expertise needed to operate monitoring equipment. Real-time personal monitoring or multi-location monitoring is, however, not a new concept. Innovations in air quality monitoring have addressed cost in the last decade. A combination of technological advancements (cheaper electronic boards and smaller light scattering sensors), public interest in air pollution, and the increased popularity of citizen science have resulted in the development and proliferation of low-cost PM_2.5_ sensors and devices. These low-cost sensors have gained in popularity for a range of uses from home and personal monitoring to citizen science and to larger scale academic research^2–4^.

The advantages of low-cost PM_2.5_ sensors for research include: (1) deployment in large numbers to increase spatial and temporal coverage; (2) ease of use and maintenance; and (3) a battery power source that permits remote or portable use [https://www.epa.gov/air-sensor-toolbox]^5–7^. In addition, they can be connected via Wi-Fi or Bluetooth technology to transmit data, sometimes in real time, to central servers and crowdsourcing platforms to share data and cover large geographic areas with extended spatial and temporal resolution. However, these simple, low-cost sensors have limitations and require routine testing and calibration prior to use in scientific studies. Much work has been done in recent years to address these limitations, and results have demonstrated that low-cost sensors generally have acceptable reliability but also technological limitations and inter-instrument variability^5–14^. A key finding of many of these studies is that important research gaps remain regarding durability and the need for calibration of individual units prior to use for research^7,10,11^.

Few studies, for example, have examined the performance of a network of low-cost sensors over an extended period. One such study showed that some PM_2.5_ sensors were relatively stable over time when tested over a year, however that study focused on measurements in outdoor environments with concentrations ranging from 6 to 41 µg/m^3^^15^. Other short-term studies have demonstrated that careful calibration of low-cost sensors demonstrate their utility for indoor measurements of PM^16,17^. In tandem, public housing authorities (PHAs) have been federally mandated to implement smoke-free housing (SFH) policies in their developments^18–20^. Despite policy implementation in July 2018, there is still some evidence of cigarette smoking within New York City Housing Authority (NYCHA) developments^18^. Stemming from a larger, quasi-experimental study evaluating the impact of SFH policies on secondhand smoke exposure in select NYCHA buildings, we utilized a network of low-cost sensors to evaluate indoor PM. This current analysis sought to assess whether rigorous calibration allows low-cost sensors to be used for indoor air quality measurements in the field for long periods of time without degradation in reliability. To achieve this objective, we repeatedly calibrated and utilized many low-cost first and second generation Airbeam PM_2.5_ sensors, over a 2-year period, to assess PM_2.5_ concentrations in urban high-rise buildings with a focus on measuring indoor tobacco smoke.

## Methods

### Generation of calibration curves for cigarette smoke

Cigarette smoke-specific calibration curves for the Airbeam 1 and 2 PM_2.5_ sensors were created in a laboratory setting via the direct comparison of the output of the low cost Airbeam sensors with simultaneous 1-min readings produced by a factory-calibrated Thermo Scientific Personal DataRAM PDR-1500 unit with a 2.5 µm inlet (Thermo Environmental Instruments, Waltham, MA). The PDR-1500 unit is a widely used instrument and shown to be reliable from previous studies^21–29^. Over the course of the 2-year period, our low-cost sensors were calibrated four times using the same PDR-1500 unit. We took pre and post weight measurements of the internal filter within the PDR-1500 unit to calculate the gravimetric concentration which allowed for the calibration of the real-time readings. The Airbeam 1 and 2 devices utilize two low-cost sensors: The Shinyei PPD60PV and Plantower PMS 7003 infra-red light scattering particle sensors, respectively. The PDR 1500 unit was zeroed with particle-free air prior to each run.

To perform the calibration, 8–12 Airbeam units were placed into an airtight stainless-steel chamber, where temperature is room temperature and humidity matches the building’s at below 50%, with access ports permitting the introduction of cigarette smoke or HEPA filtered air. The PDR-1500 was connected to a sampling port for measuring the PM_2.5_ concentrations inside the chamber. This instrument has both an inlet and outlet where tubes are connected to inject cigarette smoke into the chamber; the PDR-1500 was not placed inside the chamber to prevent contamination resulting from its enclosure with cigarette smoke. A smoking machine (Borgwaldt, Hamburg, Germany) was used to inject fresh mainstream cigarette smoke using 3R4F reference cigarettes into the chamber until the PDR-1500 registered a particle mass concentration greater than 1000 µg/m^3^. A high concentration value such as 1000 µg/m^3^ exceeds the upper limit for PM_2.5_ values for both low-cost particle sensor types. Airbeam 1 and Airbeam 2 sensors have different saturation points at 80 µg/m^3^ and 200 µg/m^3^, respectively (i.e., the light scattering derived PM_2.5_ output plateaus), ensuring the decreasing PM_2.5_ calibration curve would begin above their detection ceiling (approximately 180 µg/m^3^ and 800 µg/m^3^, respectively). After cigarette smoke generation was stopped, the sample pump and internal filter of the PDR-1500 slowly removed cigarette smoke from the chamber which was replaced by HEPA-filtered room air. The resulting time-dependent decrease in PM_2.5_ was used to develop the calibration curve. The start times of the Airbeam units and PDR-1500 particulate matter readings were synchronized, and the 1 min outputs were recorded beginning above the nominal upper detection limit and continued until the PDR-1500 values stabilized in the low single digit µg/m^3^ range. Each run lasted approximately one hour.

Readings from each Airbeam (X-axis) were matched by synchronized timestamp with the corresponding values from the PDR-1500 (Y-axis). Using Excel, a unique calibration equation for each Airbeam unit was calculated by linear regression up to 80 µg/m^3^ which was the expected upper limit for indoor PM_2.5_. Polynomial regression models were also generated; however, the output of these models was linear up to 80 µg/m^3^, which strengthened our decision to use linear models. Each unique equation and accompanying R value was recorded and assigned to the unit by serial number. Because of differences in the sensor type’s output, for consistency, we calculated calibration coefficients using the total PM reading from Airbeam 1 sensors, and the PM_10_ output from Airbeam 2 sensors. There is no physical cutoff point within Airbeam 2 sensors to differentiate between PM_2.5_ and PM_10_, so we used the highest value reported in the Airbeam to approximate PM_2.5_ from the PDR-1500 unit. The PM_10_ values from the Airbeam 2 sensors aligned closely with the corresponding values from the PDR-1500 instrument, which strengthened our decision to use PM_10_ output rather than PM_2.5_ output from the Airbeam 2 sensors to approximate PM_2.5_ from the PDR-1500. Both sensor types use an algorithm based on an internal equation to generate PM output; Airbeam 1 sensors do not have the split for PM_1_, PM_2.5_ and PM_10_ values. The calibration coefficient for cigarette smoke was developed as a multiplication factor to correct the Airbeam PM_2.5_ output and calculated as:

$$Calibration\;Coefficient = {\text{slope}}\;{\text{of}}\;{\text{ the}}\;{\text{PDR}} - {15}00 \, \left( {\text{Y - axis}} \right) \, \;{\text{vs}}\;{\text{Airbeam }}\left( {\text{X - axis}} \right)\;{\text{calibration}}\;{\text{curve}}$$

To assess the effect of particle composition on the calibration curve, the Airbeam devices were also calibrated using airborne particles in the NYC subway system. As in the cigarette smoke calibration procedure, the output of four Airbeam 1’s and four Airbeam 2’s was compared to the PDR 1500 PM_2.5_ output and a calibration coefficient was calculated for subway PM_2.5_.

### Field sampling periods

We calibrated 51 low-cost particle sensors (Airbeam 1 generation N = 29; Airbeam 2 generation N = 22) at 4 different timepoints over a 2-year period spanning from 2019 to 2021. After each laboratory calibration, the Airbeam units were deployed in a large, natural experiment evaluating the impact of new smoke-free housing (SFH) policies on air quality in public housing units every 6 months^18,30^. Due to the onset of the COVID-19 pandemic, we were unable to perform Airbeam sensor calibration from April-September 2020. A technician-based calibration error for select Airbeam 2 sensors only, from December-March 2021, led to their exclusion from data analysis at that timepoint. The final calibration timepoint was collected for all 51 Airbeam sensors from May–September 2021 to obtain a final calibration coefficient.

### Data analysis

We descriptively tabulated the mean (SD) calibration coefficients at four different 6-month timepoints over a 2-year period from 2019 to 2021 for the two different Airbeam sensor types. We performed independent t-tests to measure statistically significant differences in calibration coefficient means between particle sensor types, and characterized the between-and-within variability for calibration coefficient measurements. Because the light scattering properties of airborne particles are influenced by particle composition, we compared the mean (SD) calibration coefficients for cigarette smoke and subway PM_2.5_ using an independent t-test. Lastly, we used a difference-in-difference (DID) approach to compare within-group changes between Airbeam 1 and Airbeam 2 sensors across four different calibration timepoints. Regression models included fixed effects for particle sensor type (Airbeam 1 vs Airbeam 2 sensors) and data collection timepoints (12, 18, 30 and 36 months post-SFH policy implementation^13^). We adjusted for the clustering of individual Airbeam IDs and repeated measures overtime. Model-based mean differences with 95% confidence intervals were calculated for each particle sensor type over time. *P*-values were reported after implementation of the independent t-tests, with a significance level set at *p* < 0.05, using a two-sided test. All analyses were performed using SAS statistical software, version 9.4 (SAS institute).

We examined the individual time trends in calibration coefficient measurements for low-cost particle sensors over a 2-year period, grouped by particle sensor type (Supplemental Figure S1), and descriptively categorized all low-cost particle sensors that were taken out of circulation over the 2-year period (Supplemental Table S1). We then examined the correlation between the number of unique instances of use for individual Airbeam sensors, and their final calibration coefficients at the end of the 2-year period (Supplemental Table S2 and Supplemental Figure S2).

## Results

### Sample characteristics

We conducted a descriptive characterization of the mean (SD) calibration coefficients at four different timepoints over a 2-year timeframe from 2019 to 2021 (Table 1). At our first timepoint, our sample included a total of N = 56 Airbeam 1 sensors and N = 24 Airbeam 2 sensors. We observed more equipment failure over time in Airbeam 1 sensors (n = 27 out of 56, failure rate 48.2%) than in Airbeam 2 sensors (n = 2 out of 24, failure rate 8.3%). These equipment failures occurred for a variety of reasons including, but not limited to cockroach infestations, not recording data properly (i.e., inconsistent relative humidity, temperature, or PM outputs), reading null values in PM measurements, and failure during calibration (Supplemental Table S1). As a result, our effective sample size decreased to N = 37 Airbeam 1 sensors and N = 21 Airbeam 2 sensors at the second timepoint, and N = 29 Airbeam 1 sensors and N = 22 Airbeam 2 sensors at the third and fourth timepoints. We thus restricted the analyses to the N = 29 Airbeam 1 sensors and N = 22 Airbeam 2 sensors available across all 4 calibration time points. We performed a secondary analysis to include all data points, both from units that performed well and from units that failed, and found no significant effect on outcome (see Supplement Table S3). The PM_2.5_ concentration readout of Airbeam PM_2.5_ sensors was less than that of the PDR-1500 reference instrument at each calibration timepoint.

**Table 1:** Descriptive characterization of calibration coefficient measurements among two low-cost particle sensor types over a two-year timeframe, 2019–2021.

Time frame	Airbeam 1 (N = 29)				Airbeam 2 (N = 22)				*p*-value
	Mean	SD	Min	Max	Mean	SD	Min	Max	
TimePoint 1	1.43	0.44	0.96	2.64	1.56	0.26	1.23	2.07	0.21
TimePoint 2	1.14	0.22	0.65	1.69	1.58	0.26	0.97	2.02	< 0.0001
TimePoint 3	1.19	0.34	0.63	2.24	–	–	–	–	–
TimePoint 4	0.87	0.20	0.55	1.23	0.96	0.27	0.74	1.89	0.2

*SD* standard deviation.

### Between-and-within variability in calibration coefficients for low-cost particle sensor types

**Figure 1:**
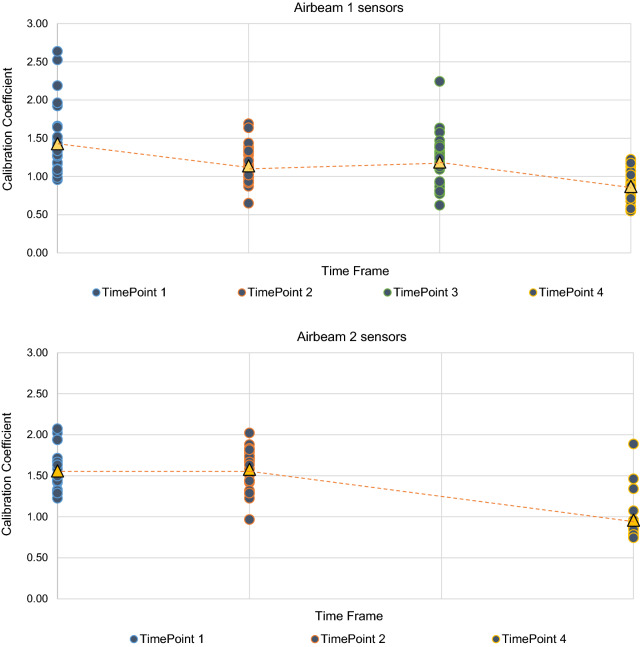
Between-and-within variability for calibration coefficient measurements over a 2-year timeframe: low-cost Airbeam 1 and Airbeam 2 particle sensors.

On an individual unit basis, we observed a high degree of inter-sensor variability in calibration coefficients across both low-cost particle sensor types over a 2-year timeframe (Fig. 1). There was a notable decline in Airbeam calibration coefficients consistent across both low-cost particle sensor types, with values trending downward to below one at the final calibration timepoint. Inter-monitor variability was high in Airbeam 1 sensors at the first calibration timepoint and in Airbeam 2 sensors at the fourth calibration timepoint. During the second calibration timepoint, the mean (standard deviation (SD)) calibration coefficient for Airbeam 1 sensors was lower compared to Airbeam 2 sensors [M(SD) = 1.14(0.22) vs. M(SD) = 1.58(0.26), *p* < 0.0001] (Table 1). Because of the technical errors in the Airbeam 2 calibrations, a calibration coefficient mean was determined only for Airbeam 1 sensors (1.19 (0.34)) during the third calibration timepoint.

### Least square mean differences in calibration coefficients for low-cost particle sensor types

We conducted a DID model approach for repeated measures and characterized the least square mean differences [(MD (95% CI)] spanning from 2019 to 2021, for each low-cost particle sensor type (Table 2). Among Airbeam 1 sensors, the degree of inter-monitor change over time was statistically significant across all four time points, apart from change between the second and third timepoints [(-0.05 (−0.23, 0.14)] *(p* = *0.6).* Among Airbeam 2 sensors, the degree of inter-monitor change over time was only statistically significant between the second and fourth timepoints [(0.62 (0.46, 0.78)] and between the first and fourth timepoints [(−0.60 (−0.76, −0.44)], but not statistically significant between the first and second timepoints [(0.02 (−0.14, 0.18)] *(p* = *0.8).* We calculated the inter-monitor change over time among three timepoints only for Airbeam 2 sensors due to a calibration malfunction from December-March 2021.

**Table 2:** Characterization of the least square mean differences for each low-cost particle sensor type over a two-year timeframe, 2019–2021.

Effect	Mean difference (95% CI) Airbeam 1 sensors	*P-*value	Mean difference (95% CI) Airbeam 2 sensors	*P-*value
Timepoint 1 to 2	−0.29 (−0.42, −0.15)	< *0.0001*	0.02 (−0.14, 0.18)	*0.79*
Timepoint 1 to 3	−0.24 (−0.41, −0.07)	*0.007*	–	*–*
Timepoint 1 to 4	−0.56 (−0.70, −0.42)	< *0.0001*	−0.60 (−0.76, −0.44)	< *0.0001*
Timepoint 2 to 3	−0.05 (−0.23, 0.14)	*0.63*	–	–
Timepoint 2 to 4	0.28 (0.14, 0.42)	*0.0002*	0.62 (0.46, 0.78)	< *0.0001*
Timepoint 3 to 4	0.32 (0.14, 0.50)	*0.0004*	–	*–*

### Comparison of calibration coefficients for cigarette smoke versus subway particulate matter

Because particle composition can affect light scattering properties, we characterized the comparison of calibration coefficient mean differences by particulate matter from two different sources at a single timepoint (Table 3), for cigarette smoke and particulate matter present in subway stations. The calibration coefficients, resulting from a pooled analysis grouped by calibration method, of 1.79 (0.76) for cigarette smoke and 1.22 (0.39) for subway PM_2.5_ were not statistically different (*p* = *0.078*).

**Table 3:** A comparison of calibration coefficient mean differences by particulate matter source composition: an individual and pooled analysis across all units at a single timepoint.

Individual unit	Calibration using cigarette smoke Mean	Calibration using subway PM Mean	Absolute mean difference
1	1.23	0.84	0.39
2	1.30	0.99	0.31
3	2.07	0.82	1.25
4	0.97	1.25	0.28
5	2.61	1.33	1.28
6	2.63	1.16	1.47
7	2.58	2.04	0.54
8	0.93	1.31	0.38

	Calibration using cigarette smoke (N = 8) Mean (SD)	Calibration using subway PM (N = 8) Mean (SD)	*P*-value
*Pooled analysis by calibration method*			
	1.79 (0.76)	1.22 (0.39)	*0.08*

*SD* standard deviation.

### Correlation between unique instances of use and final calibration coefficient for individual low-cost particle sensor types

To determine if sensor usage affected Airbeam output over time, we characterized the unique instances of use (i.e., the number of 7-day indoor sampling periods that a sensor was used), and the final calibration coefficient for all 51 individual Airbeam sensors (Supplemental Table S2). We examined the correlation between the number of 7-day indoor sampling periods that an individual sensor was used, and its final calibration coefficient at the fourth calibration timepoint (Supplemental Figure S2). We did not observe a strong correlation for Airbeam 1 sensors (R^2^ = 0.16) or for Airbeam 2 sensors (R^2^ = 0.09). The slope of the curve for Airbeam 1 suggests that the more the sensors were used, the greater the deviation of its output from the PDR-1500’s output, while the Airbeam 2 curve suggested no change with an increase in usage.

## Discussion

To our knowledge, this analysis is one of the first long-term longitudinal assessments of performance and reliability of low-cost particle sensors in measuring indoor tobacco smoking. We observed a high degree of inter-sensor variability across both particle sensor types, particularly in Airbeam 1 sensors at the study’s initiation. Change in calibration coefficients over time for individual Airbeam units was detected, suggesting a degradation of low-cost particle sensors for longitudinal assessment (Supplemental Figure S1). There were also notable downward trends in calibration coefficients over time, whereas the accompanying calibration coefficient for both Airbeam 1 and 2 sensors was below 1 at the final calibration timepoint. Findings lend support to the conclusion that the routine calibration of individual Airbeam units might help to improve their utility and performance over time. Overall, Airbeam 2 particle sensors fared better than Airbeam 1 sensors, suggesting greater durability of Airbeam 2 sensors for longitudinal assessment.

Our findings suggest that low-cost particle sensors might be differentially subjected to degradation, seen in the greater loss of Airbeam 1 sensors than Airbeam 2 sensors over time. While two of these failures, and the loss of units, resulted directly from the public housing environments (i.e., roach infestation), other failures were more generally concerning for the use of non-calibrated low-cost particle sensors for longitudinal assessment of air quality. Interestingly, we did not observe a strong correlation between the unique instances of field use of sensors over the 2-year period and their final calibration coefficients measured at the fourth calibration timepoint, suggesting that low-cost sensor degradation over time might be more contingent on particle sensor type, rather than individual sensor usage.

Calibration coefficients differed modestly between cigarette smoke or subway PM (primarily combustion products and iron-rich friction particles, respectively), suggesting that the light-scattering physics of these low-cost particle sensors may slightly be affected by these two particle source types. Our finding, however, is limited to two particular particle types, and further studies are needed to assess calibration across a range of particles with different source-dependent compositions. Other researchers have observed that, in addition to particle composition, the accuracy of PM_2.5_ sensor output also depends upon particle size and humidity^13^. Thus, low-cost sensors require routine calibration in the laboratory with the PM_2.5_ and environmental conditions of interest.

Our current analysis provides a robust assessment of the longitudinal utility of low-cost particle sensors. Previous studies have measured the utility of low-cost particle sensors for PM monitoring where reference-standard equipment is not available or feasible, and for improving the study of spatially localized airborne PM concentrations^5–14^. One study conducted in the United Kingdom evaluated the performance of four models of low-cost PM sensors and examined inter-model performance across 19 different particle sensor units. Despite differences in the way each sensor type derived PM concentrations, the researchers found general agreement in PM readings across sensor types^8^. Another study evaluated the performance of two widely-used particle sensors, the Plantower PMS A003 and Shinyei PPD42NS, and developed PM calibration models for seven different metropolitan areas (i.e., Los Angeles, Chicago, New York, Baltimore, Minneapolis-St. Paul, Winston-Salem and Seattle) using a sample of 72 sensors. The authors found that good calibration models were feasible only with the Plantower PMS A003 model after running simulations for region-specific models^7^. Another study found that a Plantower PMS 1003 sensor provided reliable PM data outputs over a 13-month period^15^. Our study extended this time period to over 2 years of reliable output from Plantower PM sensors (albeit a different model), although the reproducibility of the calibration coefficients varied by individual units over time. One of the largest programs of low-cost sensor use is currently underway with the U.S. EPA’s AirNow network of low-cost PurpleAir sensors for the nationwide monitoring of wildfire-generated PM (https://www.airnow.gov/fires/using-airnow-during-wildfires/). As demonstrated in our study of Airbeam sensors, the PurpleAir sensors report PM levels that differ from more expensive and reliable monitoring instruments, but these offsets can be corrected by a ‘correction equation.’ The underlying design of the PurpleAir device is based on the fact that low cost sensors may degrade over time and therefore the PurpleAir device evaluates individual sensor degradation by continually comparing the output of two low-cost Plantower PM sensor units built into each monitoring device^31^. As such, the EPA has published guidelines on the use and performance testing of low-cost air pollution sensors (https://cfpub.epa.gov/si/si_public_record_Report.cfm?dirEntryId=350785&Lab=CEMM). Without such corrections, caution is necessary regarding the reliability of low-cost PM sensors over time.

There were several limitations to our research. Overall, the PM output of each low-cost particle sensor differed from the PM output of the widely used PDR-1500 which has an air flow regulation and infra-red laser that are far more precise than what is available in the low-cost PM sensors, suggesting a potential for under- or over-estimation of PM levels when calibration methods are not utilized. Over time, we experienced equipment failures in a significant number of sensors, particularly the Airbeam 1 generation, thus reducing our effective sample size in this calibration study. The results in our paper may fail to cover all the low-cost sensors and calibration of low-cost PM sensors is imperative. Our routine calibration and inspection of low-cost particle sensors ensured careful use for the long-term sampling of indoor tobacco smoke. Unfortunately, this type of calibration would likely be challenging for many community groups or citizen science groups that may not have access to higher quality PM monitors. There were also several strengths to our research. Our study provides a robust assessment of the utility of low-cost particle sensors among a large number of a single brand of two generations of particle sensors available for purchase and utilized in citizen science across the U.S^32^. We compared the robustness of these two low-cost Airbeam particle sensor types, as well as across two different calibration particle types. We restricted our analysis to sensors that did not provide evidence of malfunction over time, and measured calibration coefficients over a 2-year period, allowing for the assessment of the reliability of these particles for air quality monitoring.

## Conclusions

We observed modest changes in calibration coefficient measurements over a 2-year timeframe among both low-cost Airbeam particle sensor types, but in general the later generation Airbeam 2 model was more reliable, suggesting that specific particle sensors may yield better longitudinal consistency. While we did observe a degree of inter-monitor variability, changes in calibration coefficient measurements were relatively consistent across Airbeam 1 and 2 sensors. Finally, while not significant, we observed a modest difference in calibration coefficients when using cigarette smoke and subway PM as the calibration PM. As noted by our results and that of other researchers, low-cost PM sensors can provide reliable and consistent air quality data but regular calibration of the monitors is necessary to optimize their utility.

## Supplementary Information

Supplementary Information 1.

Supplementary Information 2.

Supplementary Information 3.

Supplementary Information 4.

Supplementary Information 5.

## Data Availability

Data generated or analyzed during this study can be found within the published article and its supplementary materials found in the appendices of this article.

## Abbreviations

PM: Particulate matter
NAAQs: National ambient air quality standards
SFH: Smoke-free housing
EPA: Environmental Protection Agency
PHA: Public Housing Authority
