# Supplementary material for: Feasibility of low-cost particle sensor types in long-term indoor air pollution health studies after repeated calibration, 2019–2021
Source: Sci Rep. 2022 Aug 26;12:14571. doi: 10.1038/s41598-022-18200-0 (PMC9411839; doi:10.1038/s41598-022-18200-0)
Supplement: Supplementary file 1 — Supplementary Information 1. [file 41598_2022_18200_MOESM1_ESM.docx]

**Supplemental Figure S1.** Time Trends in Calibration Coefficient Measurements for Low-Cost Particle Sensors over a Two-year Timeframe—Grouped by Sensor Type


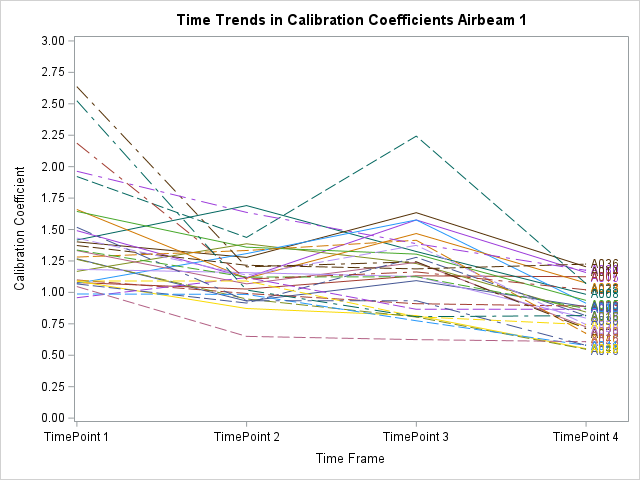


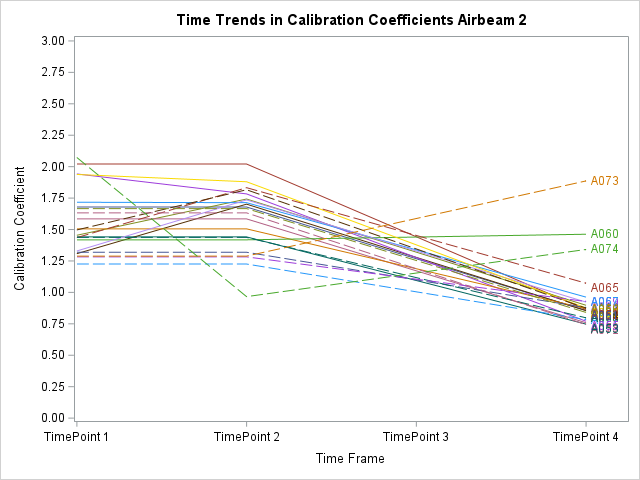


Footnote: Each line represents the time trend in calibration coefficient from TimePoint 1 to TimePoint 4 for the individual Airbeam (Airbeam 1 N=29; Airbeam 2 N=22)
