# Supplementary material for: Feasibility of low-cost particle sensor types in long-term indoor air pollution health studies after repeated calibration, 2019–2021
Source: Sci Rep. 2022 Aug 26;12:14571. doi: 10.1038/s41598-022-18200-0 (PMC9411839; doi:10.1038/s41598-022-18200-0)

Supplemental Figure S2. Correlation Between Unique Instances of Use over a 2-year Timeframe and Final Calibration Coefficient, among Individual Low-Cost Particle Sensor Types

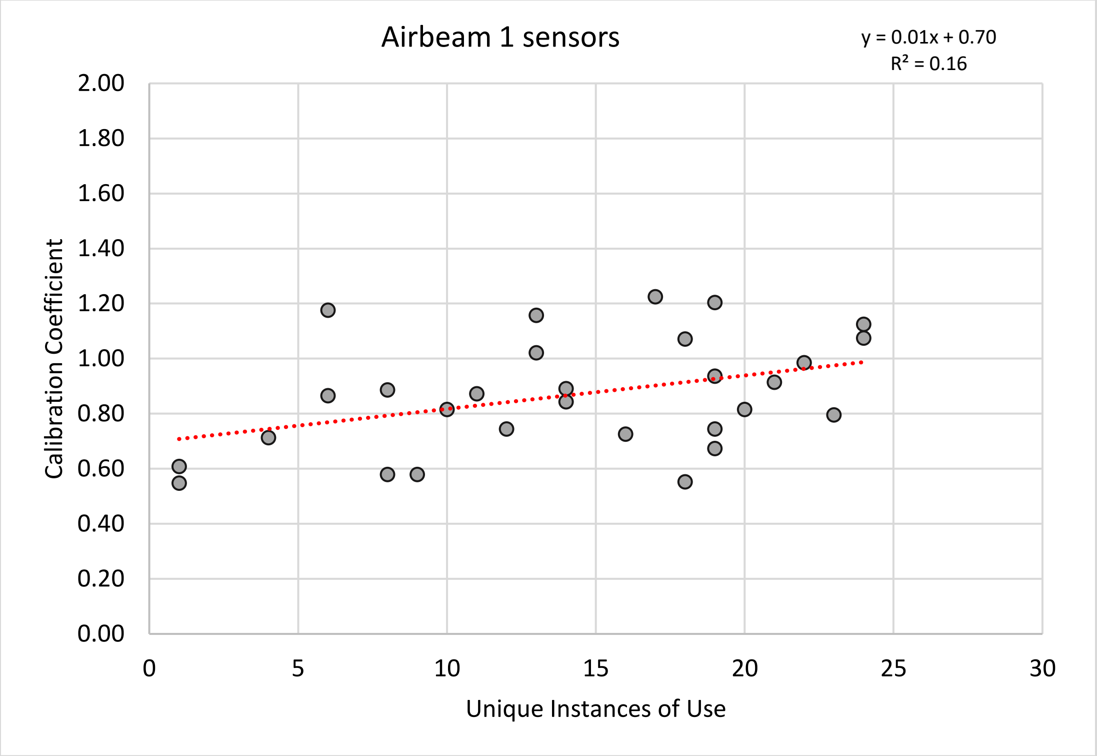

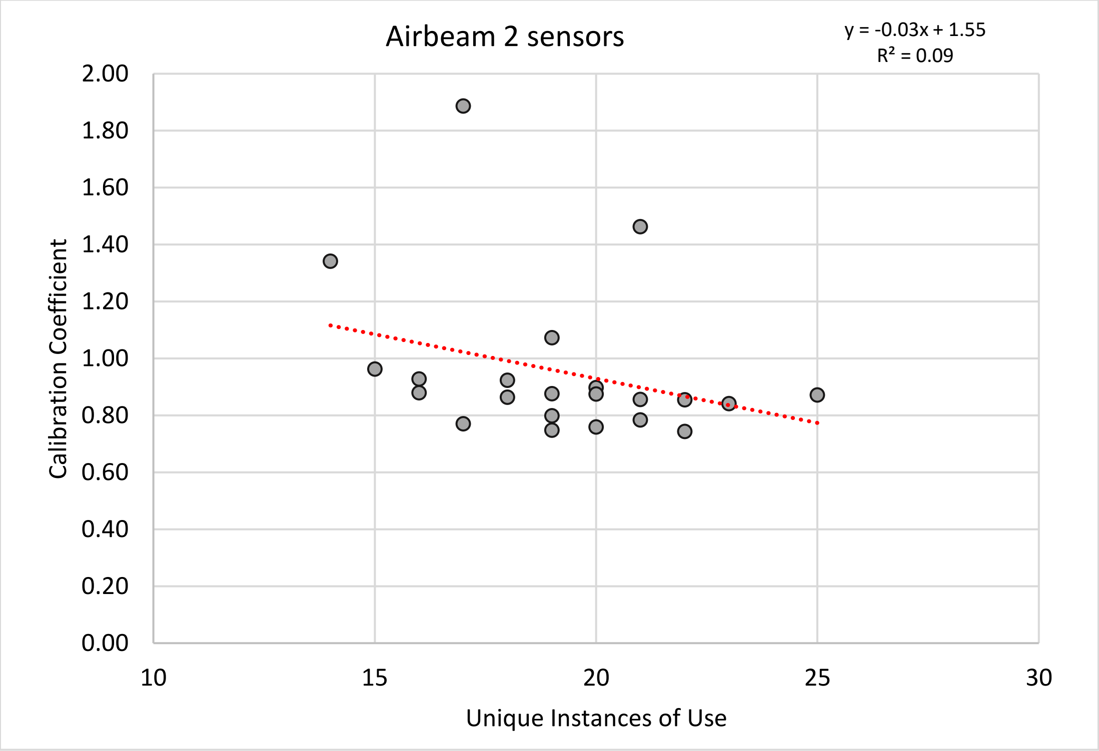

Supplement: Supplementary file 2 — Supplementary Information 2. [file 41598_2022_18200_MOESM2_ESM.pdf]
