# Supplementary material for: Feasibility of low-cost particle sensor types in long-term indoor air pollution health studies after repeated calibration, 2019–2021
Source: Sci Rep. 2022 Aug 26;12:14571. doi: 10.1038/s41598-022-18200-0 (PMC9411839; doi:10.1038/s41598-022-18200-0)
Supplement: Supplementary file 3 — Supplementary Information 3. [file 41598_2022_18200_MOESM3_ESM.docx]

**Supplemental Table S1.** List of Low-Cost Particle Sensors Taken Out of Circulation over a 2-year Timeframe

| **Low-Cost Sensor ID** | **Reason for Removal from Circulation** |
| --- | --- |
| A001 | Roach Infested |
| A002 | Roach Infested |
| A003 | Unlocatable |
| A004 | Not collecting data collection measure properly (i.e., humidity, temperature, particulate matter) |
| A005 | Noisy fan |
| A006 | Reading null values (i.e., no peak data in particulate matter measurements) |
| A009 | Failed to collect sufficient data points |
| A010 | Reading null values (i.e., no peak data in particulate matter measurements) |
| A011 | Not collecting data collection measure properly (i.e., humidity, temperature, particulate matter) |
| A012 | Reading null values (i.e., no peak data in particulate matter measurements) |
| A013 | Noisy fan |
| A017 | Broken USB port |
| A018 | Noisy fan |
| A021 | Noisy fan |
| A022 | Reading null values (i.e., no peak data in particulate matter measurements) |
| A024 | Not collecting data collection measure properly (i.e., humidity, temperature, particulate matter) |
| A027 | Noisy/ flashing green light |
| A031 | Reading null values (i.e., no peak data in particulate matter measurements) |
| A032 | Reading null values (i.e., no peak data in particulate matter measurements) |
| A034 | Failed to collect sufficient data points |
| A037 | Flashing green light |
| A038 | Noisy fan |
| A042 | Battery dependent |
| A044 | Monitor did not max out properly during the calibration run |
| A045 | Not collecting data collection measure properly (i.e., humidity, temperature, particulate matter) |
| A047 | Reading null values (i.e., no peak data in particulate matter measurements) |
| A064 * | Lost |
| A067 * | Lost |
| A075 | Reading null values (i.e., no peak data in particulate matter measurements) |

**Airbeam 2 monitor*
