# Supplementary material for: Feasibility of low-cost particle sensor types in long-term indoor air pollution health studies after repeated calibration, 2019–2021
Source: Sci Rep. 2022 Aug 26;12:14571. doi: 10.1038/s41598-022-18200-0 (PMC9411839; doi:10.1038/s41598-022-18200-0)
Supplement: Supplementary file 4 — Supplementary Information 4. [file 41598_2022_18200_MOESM4_ESM.docx]

**Supplemental Table S2.** Correlation Between the Number of Unique 7-Day Sampling Periods over a 2-year Timeframe and Final Calibration Coefficient, among Individual Low-Cost Particle Sensor Types

| **Airbeam ID** | **Number of Unique 7-Day Sampling Periods** | **Final Calibration Coefficient** | **Airbeam Type** |
| --- | --- | --- | --- |
| A000 | 8 | 0.887 | 1 |
| A007 | 24 | 1.125 | 1 |
| A008 | 22 | 0.985 | 1 |
| A014 | 19 | 1.204 | 1 |
| A015 | 13 | 1.158 | 1 |
| A016 | 14 | 0.844 | 1 |
| A019 | 21 | 0.914 | 1 |
| A020 | 16 | 0.726 | 1 |
| A023 | 24 | 1.075 | 1 |
| A025 | 19 | 0.937 | 1 |
| A026 | 23 | 0.796 | 1 |
| A028 | 18 | 0.553 | 1 |
| A030 | 20 | 0.815 | 1 |
| A033 | 14 | 0.891 | 1 |
| A035 | 18 | 1.071 | 1 |
| A036 | 17 | 1.225 | 1 |
| A039 | 6 | 0.866 | 1 |
| A040 | 1 | 0.548 | 1 |
| A041 | 9 | 0.579 | 1 |
| A043 | 1 | 0.609 | 1 |
| A046 | 19 | 0.674 | 1 |
| A048 | 11 | 0.872 | 1 |
| A049 | 19 | 0.745 | 1 |
| A050 | 12 | 0.744 | 1 |
| A076 | 8 | 0.579 | 1 |
| A077 | 13 | 1.021 | 1 |
| A078 | 10 | 0.816 | 1 |
| A079 | 4 | 0.713 | 1 |
| A080 | 6 | 1.176 | 1 |
| A051 | 21 | 0.857 | 2 |
| A052 | 18 | 0.864 | 2 |
| A053 | 19 | 0.748 | 2 |
| A054 | 22 | 0.855 | 2 |
| A055 | 17 | 0.771 | 2 |
| A056 | 20 | 0.898 | 2 |
| A057 | 15 | 0.963 | 2 |
| A058 | 20 | 0.759 | 2 |
| A059 | 25 | 0.872 | 2 |
| A060 | 21 | 1.463 | 2 |
| A061 | 18 | 0.924 | 2 |
| A062 | 19 | 0.877 | 2 |
| A063 | 16 | 0.879 | 2 |
| A065 | 19 | 1.074 | 2 |
| A066 | 19 | 0.798 | 2 |
| A068 | 20 | 0.875 | 2 |
| A069 | 16 | 0.928 | 2 |
| A070 | 23 | 0.841 | 2 |
| A071 | 21 | 0.784 | 2 |
| A072 | 22 | 0.744 | 2 |
| A073 | 17 | 1.887 | 2 |
| A074 | 14 | 1.342 | 2 |
