# Supplementary material for: Feasibility of low-cost particle sensor types in long-term indoor air pollution health studies after repeated calibration, 2019–2021
Source: Sci Rep. 2022 Aug 26;12:14571. doi: 10.1038/s41598-022-18200-0 (PMC9411839; doi:10.1038/s41598-022-18200-0)
Supplement: Supplementary file 5 — Supplementary Information 5. [file 41598_2022_18200_MOESM5_ESM.docx]

**Supplemental Table S3.**

Descriptive Characterization of Calibration Coefficient Means Among Two Low-Cost Particle Sensor Types Over a Two-Year Timeframe, 2019-2021: FULL DATASET

| **Effect** | **Airbeam 1** | | **Airbeam 2** | |  |
| --- | --- | --- | --- | --- | --- |
|  | **N obs.** | **Mean (SD)** | **N obs.** | **Mean (SD)** | ***p-value*** |
| TimePoint1 | 56 | 1.36 (0.44) | 24 | 1.53 (0.25) | *0.08* |
| TimePoint2 | 56 | 1.21 (0.35) | 24 | 1.55 (0.26) | *<0.0001* |
| TimePoint3 | 56 | 1.24 (0.39) | -- | -- | *--* |
| TimePoint4 | 56 | 1.07 (0.40) | 24 | 0.98 (0.28) | *0.32* |

**Statistical significance (p<0.05); abbreviations SD= standard deviation*

The Between and Within Sensor Variability for Calibration Coefficients Among Two Low-Cost Particle Sensor Types Over a Two-Year Timeframe, 2019-2021: FULL DATASET

trendline for calibration coefficient means across all four timepoints
